# The diagnostic value of 1.5-T diffusion-weighted MR imaging in detecting 5 to 10 mm metastatic cervical lymph nodes of nasopharyngeal carcinoma

**DOI:** 10.1097/MD.0000000000004286

**Published:** 2016-08-12

**Authors:** Guan Qiao Jin, Jun Yang, Li Dong Liu, Dan Ke Su, Duo Ping Wang, Sheng Fa Zhao, Zhi Ling Liao

**Affiliations:** a Department of Radiology; b Department of Head and Neck Surgery; c Department of Ultrasound; d Department of Pathology, Affiliated Tumor Hospital, Guang xi Medical University, Nanning, P.R. China.

**Keywords:** diffusion-weighted magnetic resonance imaging, lymph nodes, metastatic nodes, nasopharyngeal carcinoma

## Abstract

The aim of the study was to prospectively assess the diagnostic accuracy of 1.5 T diffusion-weighted imaging (DWI) for 5 to 10 mm metastatic cervical lymph nodes of patients with nasopharyngeal carcinoma (NPC). All patients with histopathologically confirmed NPC underwent DWI with 2 *b* values of 0 and 800 s/mm^2^ were enrolled. The shortest axial diameter and mean apparent diffusion coefficient (ADC) value were recorded when lymph nodes with a shortest axial diameter from 5 to 10 mm were measured. The correlation between the pathological diagnoses and mean ADC values in the benign and metastatic lymph nodes were compared using the *Z* test. Receiver operating characteristic (ROC) curve analysis was performed to evaluate the diagnostic performance of DWI. Three hundred fourteen nodes of 52 patients with NPC consisted of 46.5% (146/314) metastatic lymph nodes and 53.5% (168/314) benign lymph nodes. The mean ADC value (×10^–3^ mm^2^/s) of benign lymph nodes was (1.110 ± 0.202), which was significantly higher than that of metastatic nodes (0.878 ± 0.159) (*P* < 0.05). The sensitivity, specificity, positive predictive value, and negative predictive value, accuracy for differentiating metastatic from benign lymph nodes using a cutoff ADC value of 0.924 × 10^–3^ mm^2^/s was 83.56%, 82.74%, 80.79%, 85.28%, and 82.80%, respectively. The area under the ROC curve was 0.851 (95% confidence intervals: 0.807–0.889). This study demonstrated that DWI is helpful in detecting 5 to 10 mm metastatic lymph nodes of patients with NPC.

## 1 Introduction

The incidence rate of nasopharyngeal carcinoma (NPC) in China was 2.8/100,000 person years in men and 1.9/100,000 person years in women based on the global registry of cancer incidence. ^[1]^ NPC is distinct from the other head and neck epithelial malignancies in clinical, demographic, and geographic features, but lymphatic drainage of the nasopharynx mainly to cervical lymph nodes is similar to most other squamous cell carcinomas of the head and neck primaries. In the meantime, NPC has higher incidence for regional lymph node metastases than those of other head and neck primary tumors. A meta-analysis about regional lymph node metastases of 13 studies with 2920 NPC cases have demonstrated that the overall probability of levels II, III, IV, and V nodal involvement are 70%, 45%, 11%, and 27%, respectively. ^[2]^

Because of treatment strategies for patients with NPC, the criteria of detecting metastatic lymph nodes were mainly applied in some imaging modalities, ^[3]^ including ultrasound (US), computed tomography (CT), and magnetic resonance imaging (MRI). The shortest trans-axial diameter is the most commonly used for differentiation malignant from benign lymph nodes. ^[4]^ The shortest trans-axial diameter measurements taken using the thresholds of metastatic and benign has been put forward are 1.5 cm for levels I and II and 1 cm for levels IV to VII. In addition to this, other criteria have also been offered, [^5^ ^6^ ^7^] such as the ratio of the longest longitudinal to axial dimensions (lymph node ratio of less than 2), necrosis and extranodal spread to suggest metastases. Meanwhile, lymph nodes may still be of normal size or have lymph node ratio greater than 2 but still harbor malignant cells. Therefore, additional criteria for detecting metastatic nodes should also be sought.

Diffusion-weighted MRI is used to obtain functional information originated from Brownian motion of the water molecule proton, and offers diagnostic messages by different pathological features. Some documents have demonstrated that the apparent diffusion coefficient (ADC) by applying diffusion-weighted imaging (DWI) has been effectively used to distinguish metastatic from benign lymph node, for which could reflect tumoral microstructures. [^8^ ^9^ ^10^] However, to our knowledge, they mainly focused on larger lymph nodes (≥10 or 15 mm), the diagnostic value of DWI in detecting metastases of normal size lymph nodes in patients with NPC has not been investigated yet. Therefore, the aim of our study was to evaluate the ability of DWI for the detecting metastatic cervical lymph nodes (5–10 mm) in patients with NPC, and improve the diagnostic accuracy of normal size lymph node metastases and provide significant information for treatment planning.

## 2 Materials and methods

### 2.1 Patients

The study was approved by the institute and native ethics committee, and informed consents were also obtained by all patients. Patients with untreated suspicious NPC including the following relevant criteria for our study were enrolled, MRI examination before ultrasonographically guided fine-needle aspiration; less than 2 weeks interval between MRI examination and ultrasonographically guided fine-needle aspiration; nodes of all patients were divided into 2 groups with histologic finds as references standards: metastatic lymph nodes and benign lymph nodes; the shortest axial diameter of lymph node in the cervical regions was from 5 to 10 mm in MRI imaging; they had adequate renal function. Between August 2010 and August 2013, there were 133 patients with suspicious NPC in age ranging from 26 to 71 years (mean age, 45 ± 4.9 years) were recruited in our study. Excluded from the study were 38 patients for histologic analysis (including lymphoma, nasopharyngitis, and adenoid hyperplasia), and 30 patients for the poor MRI quality. Thirteen patients had to be excluded for flaws in procedures of ultrasonographically guided fine-needle aspiration. Diagnoses for the final study group of 52 patients (30 men and 22 women, age range 32–66 years, mean age 46 ± 6.3 years) with NPC were enrolled in our study.

### 2.2 MR imaging

All patients of MR images were performed in 1.5 T MR scanner (Avanto; Siemens, Erlangen, Germany) with a dedicated 8-channel head and neck phased array coil. The patients were conducted to lie in examination bed with the supine position, and not to move or swallow during the acquisition. All patients underwent conventional MRI, enhanced MRI and DWI from the saddle to thoracic entrance. Conventional MRI sequences included noncontrast axial turbo spine echo (TSE) T1WI and TSE-T2WI. Noncontrast axial TSE-T1WI was obtained with a repetition time (TR) of 957 milliseconds, an echo time (TE) of 19 millisecondss, a slice thickness of 5 mm, a slice space of 1 mm, a field of view (FOV) of 24 cm × 24 cm, and a matrix of 256 × 256, 1 excitations, acquisition time 1 minute 40 seconds. Noncontrast axial TSE-T2WI acquired with TR 6760 milliseconds, TE 91 milliseconds, slice think 5 mm, slice space 1 mm, FOV 24 cm × 24 cm, matrix 384 × 384, 1 excitation, acquisition time 3 minutes 58 seconds. Axial contrast T1WI (0.2 mL/kg, gadolinium DTPA, injection rate of 3.0 mL/s) was performed with TR of 957 milliseconds, TE of 19 milliseconds, a slice thickness of 5 mm, a slice space of 1 mm, FOV 24 cm × 24 cm, and matrix of 256 × 256, 1 excitation, acquisition time 3 minutes 6 seconds. DWI using single-shot, spin-echo, and echo-planar imaging (SS-EPI) sequence of transverse plane scanning: TR 3400 milliseconds, TE 100 milliseconds, FOV 23 cm × 23 cm, matrix 384 × 384, 4 excitations, slice thickness 5 mm, slice space 1 mm, acquisition time 3 minutes 56 seconds. DWI images were obtained with different gradient factors (*b* values of 0, and 800 s/mm^2^). The motion probing gradient pulses were not applied in all 3 orthogonal directions.

### 2.3 Image and data analysis

Two experienced MR physicians interpreted the MR studies together after visual inspection of the pictures comprising of transverse, sagittal, and coronal sections. For the checklist in MR images, the lymph nodes subdivided into specific levels were based on the imaging-based nodal classification. ^[4]^ Level II contains the upper jugular nodes and extends caudally from the level of the skull base to the hyoid bone. Level III contains the middle jugular nodes, with its superior extent the hyoid bone and its inferior limit the lower margin of the cricoid cartilage. Level IV contains the lower jugular lymph nodes from a superior limit at the lower margin of the cricoid cartilage to an inferior limit at the clavicle. Level V contains spinal accessory nodes situated posterior to the posterior edge of the sternocleidomastoid muscle. Lymph nodes in some sites, including level I, level VI, level VII, supraclavicular fossa, retropharyngeal, parotid, suboccipital, and facial nodes, were not analyzed, for no biopsy of these nodes was carried out after weighing the risk and benefits.

The mean ADC values of each lymph node determined as placing regions of interest by using the same workstation was used to generate ADC maps in the following procedures. First, 2 experienced MR physicians interpreted ADC after visual inspection of the sequences comprising noncontrast T1WI, noncontrast T2WI, and contrast T1WI. Second, the maximal short-axial diameter lymph nodes from level II to V in all continuous sections were viewed, and 5 to 10 mm lymph nodes were selected. Round-shaped regions of interest were carefully placed on each selected section of the ADC map including the area with the low ADC value determined with visual inspection, special care was taken not to include cystic, necrotic, or hemorrhagic regions. Then, ADC values were generated automatic by software provided by the MR imaging system manufacturer (NUMARIS/4, Siemens). To determine the shortest axial diameter in lymph nodes, a distance cursor was used to measure the diameter in the transverse plane. These tasks were performed by 2 radiologists (JGQ, 10 years of experience, SDK, 15 years of experience) who had experienced in performing MR of the cervical lymph nodes. Two radiologists had no knowledge of the clinical, histology, and worked independently without consultation with one another. The measurements obtained by 2 radiologists were averaged.

### 2.4 Statistical analysis

Statistical analyses were performed MedCalc statistical software for Windows version 9.4.2.0. Primary statistical analysis of the pooled data was compiled by mean ± standard deviation between metastatic and benign lymph nodes. Comparisons of the ADC values and diameter between metastatic and benign nodes were performed by using *Z* test. To find the optimal ADC threshold value to differentiate metastatic from benign lymph nodes, determined by maximal Youden index defined as sensitivity plus specificity minus 1, we drew the receiver operating characteristic (ROC) curve. The sensitivity, specificity, accuracy, positive predictive value (PPV), negative predictive value (NPV), and the area under ROC curves with a 95% confidence interval (CI) of the ADC for diagnosis of metastatic nodes were calculated. The Bland–Altman plot was used to verify the level of agreement between 2 observers. *P* < 0.05 was considered to indicate statistical significance.

## 3 Results

### 3.1 Lymph node levels and histopathological analysis

**Figure 1:**
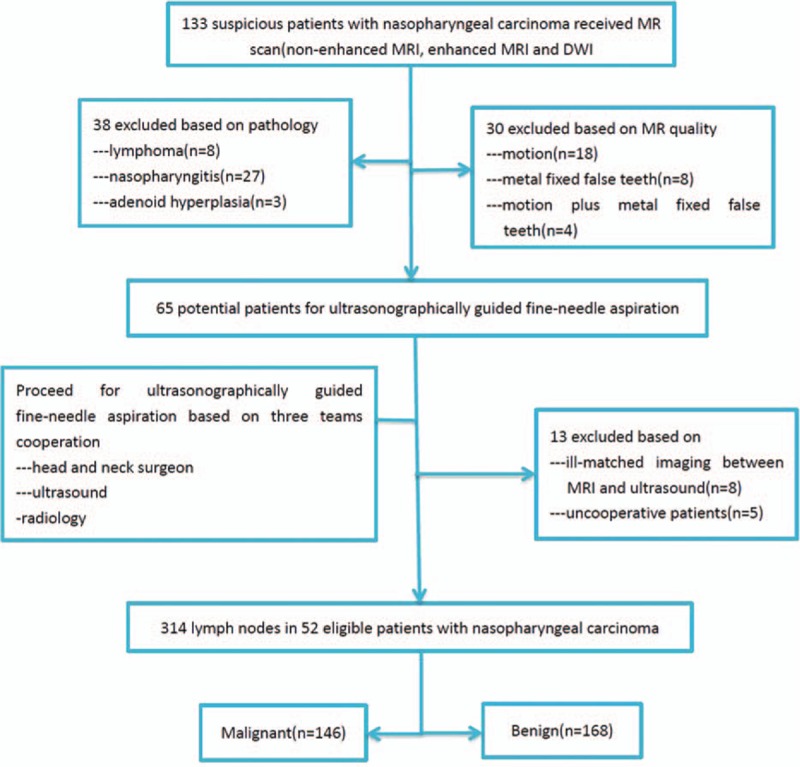
Flow chart for 314 lymph nodes of 52 patients with nasopharyngeal carcinoma fulfilled the inclusion criteria.

Three hundred fourteen lymph nodes in 52 patients with NPC in each region are shown in Table 1. Three hundred fourteen cervical lymph nodes were received by ultrasonographically guided fine-needle aspiration biopsy, 46.5% (146/314) cervical lymph nodes were metastatic, 53.5%(168/314) lymph nodes were benign (Fig. 1). The most commonly invaded regions happened in level II lymph nodes (59.1%), and rate of level III, IV, and V nodal metastasis was 31.9%, 8.3%, and16.7%, respectively.

**Table 1:**
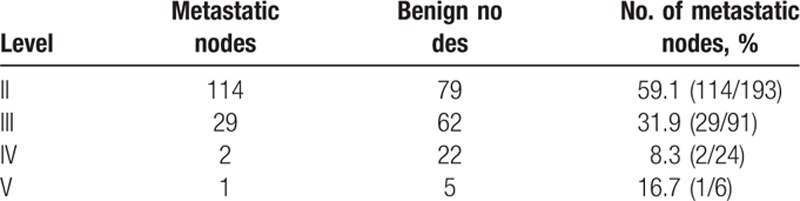
Distribution of 314 lymph nodes in 52 patients with nasopharyngeal carcinoma.

### 3.2 Diameter and ADC value of lymph node

**Figure 2:**
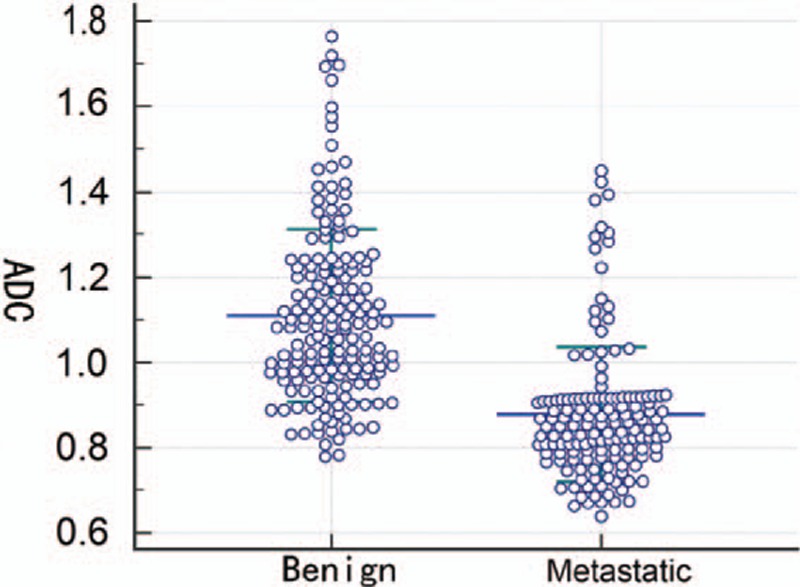
Dot plot illustrated the distribution of apparent diffusion coefficients (ADCs) of benign and metastatic lymph nodes in patients with nasopharyngeal carcinoma. Blue line and green line in dot plot represented mean and standard deviation, respectively.

**Figure 5:**
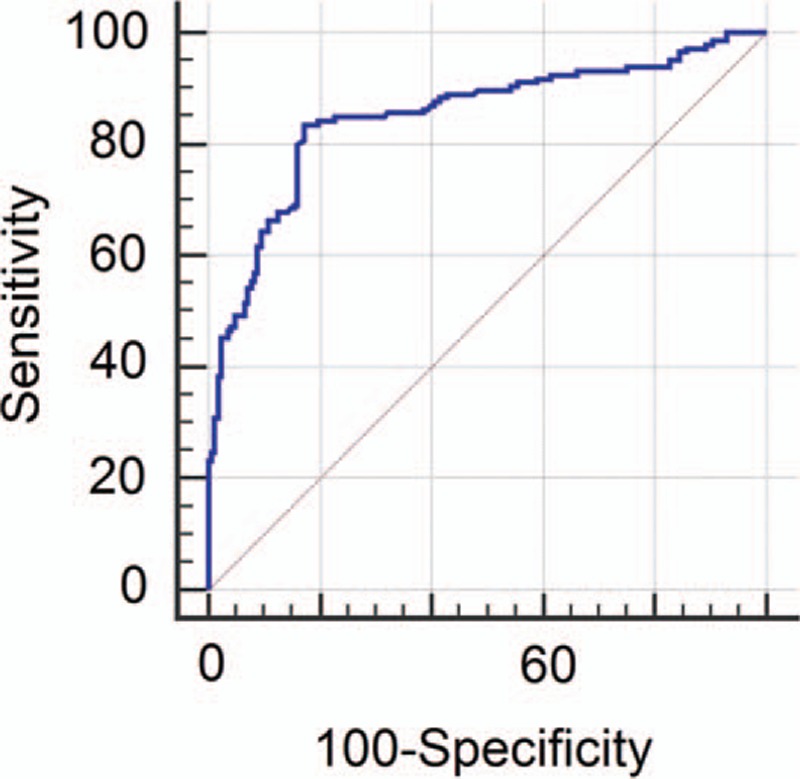
Receiver operating characteristic (ROC) curve of the ability of apparent diffusion coefficient to predict metastatic lymph nodes of patients with nasopharyngeal carcinoma. The area under the ROC curve was 0.851. The optimal threshold for detecting metastatic node was 0.924 × 10^–3^ mm^2^/s with 83.56% sensitivity and 82.74% specificity, respectively.

The results of diameter and ADC value of malignant and benign lymph nodes are shown in Table 2 and Fig. 2. Mean diameter of metastatic nodes had relatively bigger than that of benign nodes, but there had no statistic difference (*P* = 0.24). Mean ADC values in metastatic node were significant lower than that of benign nodes (*P* < 0.05) (Figs. 3A–D and Fig. 4A–D). The optimal ADC threshold value for distinguishing metastatic nodes from benign nodes was 0.924 × 10^–3^ mm^2^/s, when we optimized both sensitivity and specificity with equal weighting. For predicting metastatic nodes by the optimal ADC threshold values, the sensitivity, specificity, PPV, NPV, accuracy was 83.56%, 82.74%, 80.79%, 85.28%, and 82.80%, respectively. The area under the ROC curve of ADC values was 0.851 with 95% confidence intervals, 0.807 to 0.889 (*Z* = 15.409, *P* = 0.000) (Fig. 5).

**Table 2:**
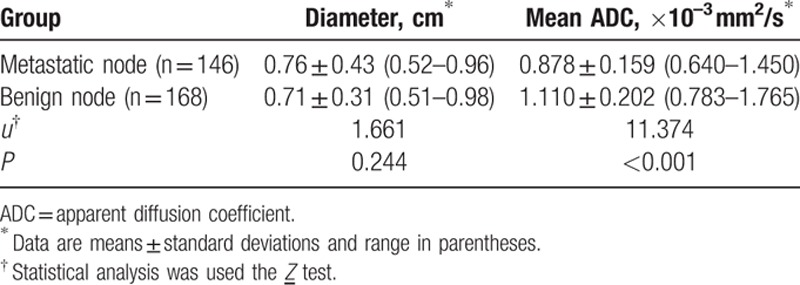
Diameter and ADC value of metastatic and benign lymph nodes in 52 patients with nasopharyngeal carcinoma.

**Figure:**
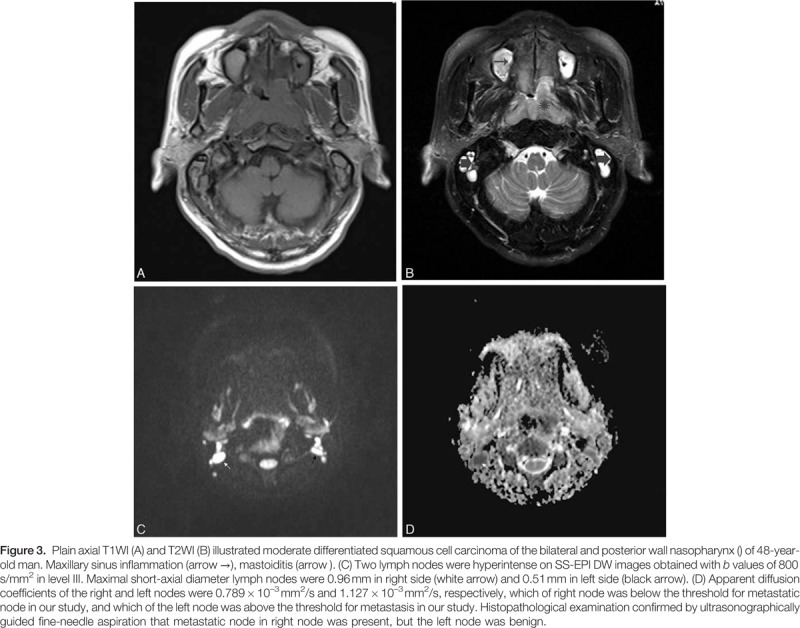


**Figure:**
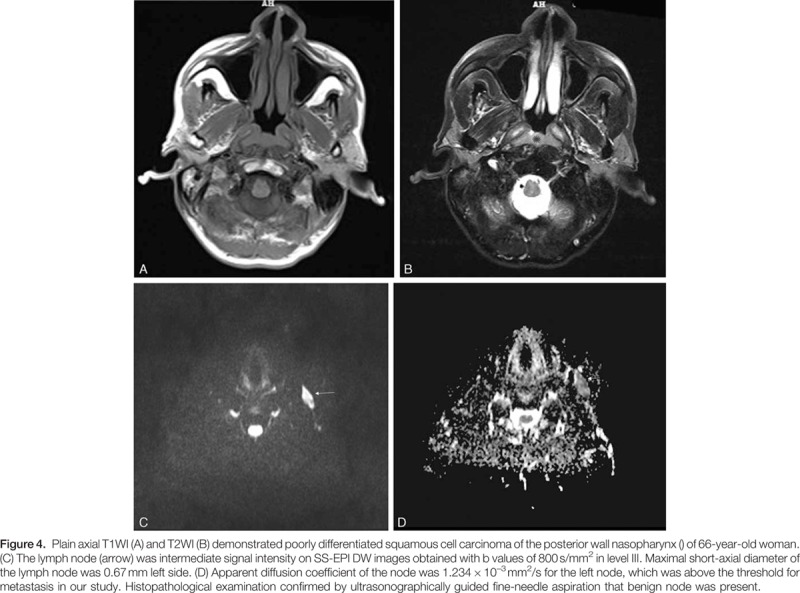


### 3.3 Test–retest variability

**Figure 6:**
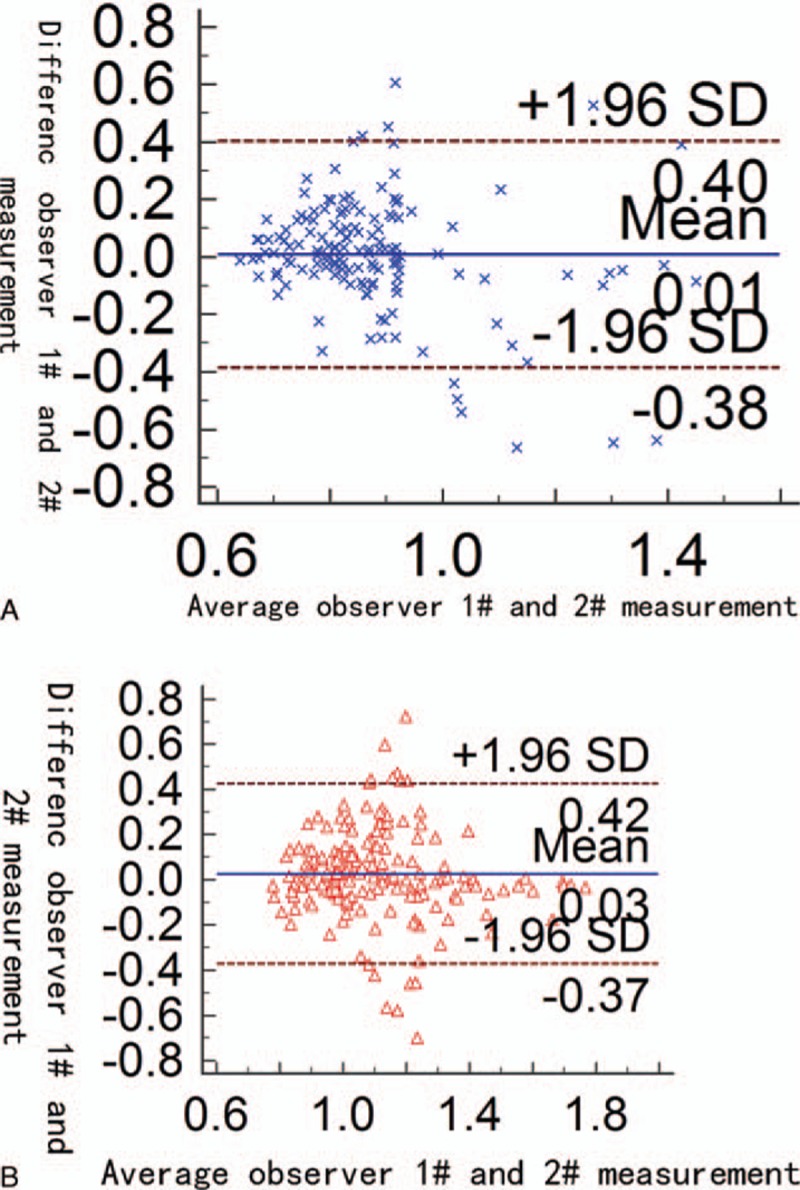
Bland–Altman plots showed observer 1 and observer 2 agreements for apparent diffusion coefficient measurements of metastatic (A) and benign (B) lymph nodes with mean difference (thick line) and 95% limits of agreement (dashed lines).

The test–retest variability of ADC value between 2 observers was assessed using the Bland–Altman plot. The Bland–Altman plot had illustrated the level of agreement between 2 observers in measurements of metastatic and benign node ADCs (Fig. 6A and B). When the mean difference of the measurements obtained by different examiners was compared, an asymmetrical distribution was observed around the mean. Relatively wide limits of agreement and relatively high bias were observed, particularly in the benign node group.

## 4 Discussion

Due to anatomic constrains and a high degree of radiosensitivity, radiotherapy has been the mainstream of treatment for NPC. ^[5]^ The results of patients with NPC are undesirable when radiation therapy alone is performed, for the minority of NPC patients with early-stage disease (I–IIa) are a minority, and the majority of cases of NPC with locally advanced stage (III–IVa) are found.[ ^11^ ^12^] There are sufficient evidences to support chemoradiotherapy should be recommended as the standard of management for locally advanced NPC in the whole world, for concomitant chemoradiotherapy in patients with local advanced NPC made a big progress in overall survival and disease-free survival for all histological types. ^[13]^ The literatures have been reported that the 5-year survival rates were about 34% to 52%, and nodal metastasis was considered as an adverse prognostic factor in patients with NPC. ^[14]^ The pathway for metastatic spread of cervical nodal chain in patients with NPC follows a typical model with a very low risk of 0.5% in skip nodal metastasis, level II nodes were susceptible to nodal metastases. Affected level III, level IV, and the supraclavicular fossa nodes, or extended posteriorly to involve level V nodes were occurred in sequence. ^[15]^ Nodal level was considered as the first important lymph node variables in the N classification, and the differentiation between benign and metastatic cervical lymph nodes of NPC is essential for the staging and playing a role of planning the extent of any radiation treatment field and determining the best therapy project, but remains challenging by the morphological criteria of conventional MRI examination.

Now, MRI can not only well demonstrate T-primary tumor, but also identify cervical lymph nodes. So far, the diagnosis of lymph node metastases for radiologist and radiation oncologists is based mainly on size criteria when interpreting cervical adenopathy on MR images of patients with NPC, [^16^ ^17^ ^18^] such as malignancy was usually suggested when the shortest axial diameter of cervical nodes in level I–VII was at least 10 mm, a cluster of more than 3 borderline size nodes was another imaging reference for metastasis, central necrosis reliably identified in tumor foci larger than 3 mm was considered as 100% specificity of diagnosis on node metastasis, and extracapsular spread was usually indicators of metastasis in large nodes, etc. However, nonenlarged nodes may harbor malignancy, whereas reactive nodes may be enlarged. Consequently, an accurate noninvasive method to diagnose lymph nodes of borderline size without nodal necrosis or extracapsular spread would be highly desirable.

From our limited date, we had demonstrated that the rate of metastatic and benign cervical lymph nodes were 46.5% and 53.5%, and percentage of involved regions in level II to V lymph nodes was 59.1%, 31.9%, 8.3%, and 16.7%, respectively. Based on the previous documents, the incidences of involved nodes patients with NPC ranged from 64.1% to 84.2 %, and the rates of nodal metastases in level II, level III, level IV, and level V ranged from 70% to 97.9%, 42.9% to 46%, 9.5% to14.3%, 13.7% to 27%, respectively.[ ^14^ ^19^ ^20^] Compared with those documents, our results about rate of metastatic cervical lymph nodes were relative low. Some factors may be responsible for the disparity, such as pathological reference by ultrasonographically guided fine-needle aspiration biopsy, the lymph node size of 5 to 10 mm, and different staging. On the other hand, our result of pathway for metastatic spread has demonstrated a decreasing frequency of nodal metastases along the jugular chain groups descending toward the supraclavicular fossa, which was consistent with cervical node involvement in NPC generally distributed in an orderly manner down the neck to the supraclavicular fossa.[ ^14^ ^19^ ^20^ ^21^]

Our study have revealed that mean ADC values in metastatic node were significant lower than that of benign nodes by DWI applying b values of 0, 800 s/mm^2^. Compared with recent articles about differentiation of metastatic and benign cervical nodes by DW imaging, there were some divergences and similarities. Li et al ^[22]^ have showed that the mean ADC between metastatic and nonmetastatic retropharyngeal nodes was statistically significant differences by DWI (*P* < 0.001). Holzapfel et al ^[23]^ have also demonstrated that mean ADC values (×10^–3^ mm^2^/s) of benign cervical lymph nodes (1.24 ± 0.16) were significant higher than that of metastatic lymph nodes (0.78 ± 0.09). Similarly, Abdel Razek et al ^[24]^ have indicated that the mean ADC value of metastatic (1.09 ± 0.11 × 10^–3^ mm^2^/s) was significantly lower than that of benign (1.64 ± 0.16 × 10^–3^ mm^2^/s) cervical lymph nodes (*P* < 0.05). Our results and above mentioned researches have demonstrated that ADC values could distinguish metastatic from benign nodes by DWI, for metastatic tumor to the regional lymph nodes is associated with lymph nodal alterations of water diffusivity and microcirculation (such as distinction of extracelullar extravascular space, cellular polymorphism, mitosis, and the number of cells), and high signal intensity in the DWI (irrelevant to the *b* value) that corresponds to low ADC values and reflects the altered changes in the proportion of extracellular to intracellular water protons were happened in the nodal metastases.[ ^4^ ^25^] However, Lim et al ^[26]^ had reported that measuring mean ADC could not allow differentiating benign from metastatic cervical lymph nodes and small nonnecrotic lymph nodes in patients with head and neck cancer, for the proportion of metastatic foci within a small metastatic lymph node without necrosis or morphological change is relatively small, and that small and dispersed metastatic deposits in an otherwise normal lymph node might not create sufficient architectural change to affect the mean ADC value.

We found the ADC threshold value of metastatic cervical lymph nodes in patients with NPC had been researched on small lymph node (5–10 mm). We believe that identified metastatic nodes of patients with NPC are more important than missing benign nodes, which is essential for the staging of NPC and its therapy planning. When we take 0.924 × 10^–3^ mm^2^/s as the optimal ADC threshold value of metastatic cervical lymph node, the diagnostic sensitivity, specificity, PPV, NPV, accuracy was 83.56%, 82.74%, 80.79%, 85.28%, and 82.80%, respectively. The result indicated that DWI had a better diagnostic efficiency for discriminating small metastatic from benign lymph nodes than that of other imaging modalities. ^[16]^ Although DWI are promising for distinguishing malignant from benign nodes, but the benefit of DWI for detecting nodal metastases, as compared with the other imaging techniques, still has to be addressed. ^[27]^

There are some limitations to our study. First, the number of metastatic lymph nodes in patients with NPC remains controversial, for about 11.6% false-negative findings of ultrasonographically guided fine-needle aspiration biopsy was reported. ^[28]^ Second, for some selected nodes were bias in ADC measures, lymph nodes smaller than 5 mm may be malignant for some microscopic metastases, which could not be obtained by ultrasonographically guided fine-needle aspiration biopsy. ^[29]^ Third, the major limitation of this work was that it was a node-by-node analysis and not station-by-station analysis. Fourth, due to small sample size and overlap in data between metastatic and benign nodes, validation of the proposed cutoff values of ADC values needs to be done in a larger scale trial. In addition, the interrater agreement was assessed using the Bland–Altman plot, and unsatisfactory results were obtained. Reproducibility of ADC measurement affected the efficiency of DWI for distinguishing metastatic from benign lymph nodes in patients with NPC.

In conclusion, we have demonstrated that DWI could detect metastases in 5 to 10 mm lymph nodes of patients with NPC. Therefore, DWI may be clinically useful in playing a role of planning the extent of radiation treatment field, and determine the best therapy project in patients with NPC.
